# The association between high birth weight and the risks of childhood CNS tumors and leukemia: an analysis of a US case-control study in an epidemiological database

**DOI:** 10.1186/s12885-017-3681-y

**Published:** 2017-10-16

**Authors:** Long Thanh Tran, Hang Thi Minh Lai, Chihaya Koriyama, Futoshi Uwatoko, Suminori Akiba

**Affiliations:** Department of Epidemiology and Preventive Medicine, Kagoshima University Graduate School of Medical and Dental Sciences, 8-35-1, Sakuragaoka, Kagoshima, 890-8544 Japan

**Keywords:** Childhood cancer, Leukemia, CNS tumors, Birth weight

## Abstract

**Background:**

High birth weight (BW), 4000 g or larger, is an established risk factor for childhood leukemia. However, its association with central nervous system (CNS) tumor risk is yet unclear. The present study examined it, analyzing data obtained from a case-control study conducted among three states from the US. The association with childhood leukemia risk was also further examined.

**Methods:**

In this study, a data set provided by the Comprehensive Epidemiologic Data Resource was analyzed with an official permission. The original case-control study was conducted to examine the association between paternal preconception exposure to ionizing radiation and childhood cancer risk. Cases with childhood cancer were mainly ascertained from local hospitals, and controls were selected, matched with birth year (1-year category), county of residence, sex, ethnicity and maternal age (+/−2 years). Since the ID numbers were unavailable, conventional logistic analyses were conducted adjusting for those matching variables except for the county of residence. In addition to those variables, gestational age, age at diagnosis and study sites as covariables were included in the logistic models.

**Results:**

Analyzed subjects were 72 CNS tumor cases, 124 leukemia cases and 822 controls born from 1945 to 1989. The odds ratios (ORs) of CNS tumor risk for children with low BWs (<2500 g) and high BWs (>4000 g) were 2.0 (95% confidence interval [CI]) = 0.7, 5.9) and 2.5 (95%CI = 1.2, 5.2)], respectively. When high-BW children were restricted to those who were large for gestational age (LGA), the OR for high-BW children remained similar (OR = 2.7; 95%CI = 1.1, 6.2). On the other hand, the ORs of leukemia risk for children with low and high BWs were 0.8 (95%CI = 0.2, 3.0) and 1.4 (95%CI = 0.7, 2.6), respectively. In the normal range of BW (2500–4000 g), higher BW was positively associated with CNS tumor risk (beta = 0.0011, p for trend = 0.012). However, the association with leukemia risk was not significant (beta = −0.0002, p for trend = 0.475).

**Conclusion:**

High-BW and LGA children had an elevated childhood CNS tumor risk. In the normal BW range, the BW itself was positively related to CNS tumor risk. No significant association between BW and childhood leukemia risk was observed in this study.

**Electronic supplementary material:**

The online version of this article (10.1186/s12885-017-3681-y) contains supplementary material, which is available to authorized users.

## Background

A recent study, reported by Steliarova-Foucher et al. [1], revealed that the incidence of childhood cancer from 2001 to 2010 has increased since the 1980s in most parts of the world. The most common cancers among children are leukemia and central nervous system (CNS) tumors. According to a recent study conducted in the US, a significant upward trend in the incidence rate of acute lymphocytic leukemia (ALL) was noticed in children aged 5 to 9 years between 2000 and 2010; however, the incidence rates of CNS tumors remained stable [2]. For children aged 10 to 14 years, however, the incidence rates of both ALL and CNS tumors increased significantly [2]. A few genetic syndromes and ionizing radiation are established risk factors for both childhood leukemia and CNS tumors [3, 4]. High birth weight (BW), 4000 g or larger, is also known to be a risk factor for childhood leukemia, especially ALL [5–8]. However, its association with childhood CNS tumor risk is yet unclear [5, 6, 9, 10].

In a large case-control study of children younger than 5 years of age, conducted in Texas, the US, the leukemia risk was elevated among those with high BWs (Odds ratio [OR]) = 1.36; 95% confidence interval [CI] = 1.10, 1.69). However, the CNS tumor risk was not evidently increased among them (OR = 1.14; 95%CI = 0.83, 1.56) [6]. Similar results were obtained by a German study. High-BW children had ORs of 1.41 (95%CI = 1.08, 1.84), 1.56 (95%CI = 0.88, 2.79) and 1.34 (95%CI = 0.97, 1.85), respectively, for ALL, acute myeloid leukemia (AML) and CNS tumors when compared to normal-BW children [11]. However, it should be noted that gestational age (GA) was not adjusted in those studies. Another large case-control study, conducted in California, which focused on CNS tumors reported a GA-adjusted OR of 1.12 (95%CI = 0.91, 1.38) [9]. In a population-based case-control study conducted in four Nordic countries, the ORs of ALL, AML and CNS tumors were 1.3 (95%CI = 1.1, 1.5), 1.5 (95%CI = 1.3, 1.8) and 1.3 (95%CI = 0.85–2.0), respectively, when children with BWs of 4500 g or larger were compared to those with 3000–3500 g, adjusting for GA [12]. Taken together, the leukemia risk was increased by 30% to 50% even after the adjustment for GA. In the case of CNS tumor risk, the association appears to be weaker.

Regarding the effect of BW itself, several studies have investigated its effect on the risks of childhood leukemia and CNS tumors. Previous studies in Texas [6] and California [13] consistently found that an each 1000-g increase in BW was associated with leukemia risk: the ORs (95%CI) were 1.28 (1.12–1.44) and 1.11 (1.01, 1.21), respectively. On the other hand, the association of BW with CNS tumor risk in those states was not statistically significant: ORs were 1.17 (95%CI = 0.98, 1.40) [6], and 1.11 (95%CI = 0.99, 1.24) [9], respectively.

Longer GAs are also suspected to be a risk factor for CNS tumors. A French study [14] reported that children with longer GAs (41 weeks or longer) were at an increased CNS tumor risk (OR = 1.4; 95%CI = 0.6, 3.3) when compared to those with the GA of 37–40 weeks, although there was no statistical significance. A Swedish study observed a similar trend in which children with the GA of 43 weeks or longer had a 1.2-fold increase of brain tumor risk (OR = 1.2; 95%CI = 0.4, 3.8) when compared to those with the GA of 38–42 weeks [15]. Only a slight increase in the CNS tumor risk was observed in the Texas study (OR = 1.07; 95%CI = 0.78, 1.47) [6]. However, the findings on the association of leukemia risk with GA were inconsistent. The Texas study reported that children with the GA of 41 weeks or longer had a slightly decreased leukemia risk (OR = 0.91; 95%CI = 0.71, 1.15) when compared to those with the GA of 37–40 weeks [6]. A contrary result was reported in a study conducted in Denmark, Sweden, Norway and Iceland, which pointed to an OR of 1.08 (95%CI = 0.90, 1.29) for longer GAs (42 weeks or longer) compared to the GA of 40–41 weeks [16].

BW is strongly related to GA [17]. Based on GA, BW can be divided into three categories: small for gestational age (SGA), appropriate for gestational age (AGA) and large for gestational age (LGA). In the Texas study, the LGA was significantly associated with an increased ALL risk (OR = 1.66; 95%CI = 1.32, 2.10), but not for CNS tumor risk (OR = 1.14; 95%CI = 0.82, 1.58) [6]. A study in California also showed no significant association between LGA and the risk of CNS tumors (OR = 1.09; 95%CI = 0.89, 1.27) [9]. In the German study, the OR of ALL was 1.45 (95%CI = 1.07, 1.97) in LGA children compared to AGA children. However, the OR for CNS tumor was not statistically significant: 1.18 (95%CI = 0.80, 1.72) [11]. In the Nordic study, LGA was related neither ALL risk (OR = 1.2; 95%CI = 0.91, 1.5) nor CNS tumor risk (OR = 1.1; 95%CI = 0.85, 1.4) [12]. Taken together, those studies suggested that LGA children may be at an elevated ALL risk. The association with the risk of CNS tumors is unlikely.

The studies described above showed no association between CNS tumor risk and SGA. The ORs in the studies of California [9], Texas [6], West Germany [11], and the Nordic countries [12] were 0.96 (95%CI = 0.75, 1.23), 0.98 (95%CI = 0.70, 1.38), 0.96 (95%CI = 0.67, 1.37) and 0.95 (95%CI = 0.77, 1.20), respectively. However, the findings on the association between leukemia risk and SGA are inconsistent. The ORs for all types of leukemia and ALL were 0.88 (95%CI = 0.68, 1.13) and 0.78 (95%CI = 0.57, 1.05), respectively, in the Texas study [6]. The ORs for ALL and AML were 1.00 (95%CI = 0.74, 1.35) and 0.89 (95%CI = 0.43, 1.83), respectively, in the German study [11], and 1.2 (95%CI = 0.96, 1.50) and 1.8 (95%CI = 1.1, 3.1), respectively, in the Nordic study [12].

We analyzed data from a case-control study which was originally conducted in the US to examine the association between paternal preconception exposure to ionizing radiation and the risk of childhood cancer, and this study found no association between them [18]. Using this dataset, we examined the association between BW and childhood cancer risk.

## Methods

### Overview of data from the CEDR database

We used data from a case-control study of childhood cancers and paternal preconception occupational exposure to ionizing radiation in counties surrounding three US Department of Energy (DOE) nuclear facilities. The data, which were obtained by the study conducted by Sever et al. [18], are available in the Comprehensive Epidemiologic Data Resource (CEDR) database through CEDR website [19] after getting an official permission. The three facilities were the Hanford (Hanford), Idaho National Engineering Laboratory (INEL) and Oak Ridge (K-25, Y-12, and X-10 at Oak Ridge laboratories). The counties selected for the study in each of 3 DOE nuclear facilities were as follows: the Benton and Franklin counties in Handford; the Bannock, Bingham, Bonneville, Buttee, Jefferson and Madison counties in INEL; and the Anderson, Knox and Roane counties in Oak Ridge. Those counties were selected, as most of the workers of the corresponding DOEs at those sites resided in them [18].

This study included 75 CNS tumor cases, 132 leukemia cases and 26 non-Hodgkin’s lymphoma cases, which were diagnosed prior to the age of 15 years, from 1957 to 1991. According to the original report [18], cases had to be born to residents of one of the study counties and be residents of one of them when their cancer was diagnosed. Cases were ascertained from each of the populations, using multiple sources (local primary care hospitals, regional referral hospitals, cancer registries and death certificates), as population-based cancer registries were unavailable in those areas during the period of 1957–1991. The controls analyzed in the present study (*N* = 1047) were matched based on year of birth (1-year category), county of residence, sex, ethnicity and maternal age (+/−2 years). The controls in the original study consisted of children identified from birth certificates. In the case of Hanford, the birth certificate controls were selected from a computer file provided by the Technical and Data Services Section, Center Health Statistics, Washington State Department of Health [18]. Server et al. identified all the births that matched each case on the basis of the year of birth, race, sex and maternal age. A file of potential controls was developed; this included all the births matching each case.

For all the cases, information on diagnosis and cause of death was abstracted from hospital records, tumor registries and death certificates in the original study. Sever et al. [18] stated in their report that "each source was utilized to provide as complete an ascertainment as possible". Pathological reports were reviewed to obtain the most accurate histopathological data.

Demographic information including sex, ethnicity, year of birth and address at the time of the diagnosis was abstracted from birth certificates or electronic birth files. Information on parental employment was collected from records at the DOE sites. Information on pregnancy (parity, date of the mother’s last menstrual period, initiation of prenatal care, viral infections during pregnancy and X-ray during pregnancy), delivery (breach or other malpresentation and clinical estimation of GA), and newborn characteristics (plurality, BW and congenital malformation) was obtained from medical records [18].

### Inclusion/exclusion criteria

In our study, we excluded children in whom information on BW, GA and year of diagnosis was lacking. Those whose ethnicities were categorized as others or unknown were also excluded. Non-Hodgkin’s lymphoma cases were not used because the number of cases was few for statistical analysis. After excluding ineligible subjects, the number of eligible subjects for CNS tumor cases, leukemia cases and controls used in statistical analysis were, 72, 124 and 822, respectively.

### Statistical analysis

We analyzed the association between BW and the risks of CNS tumors and leukemia, using a conventional logistic model [20]. All *p* values were two-sided and calculated, using the likelihood ratio test. The p values for trend were calculated, using continuous variables. Data analyses were performed, using Software Stata 14.0.

In the original study, the cases and controls were matched according to the year of birth (1 year category), county of residence, sex, ethnicity (black or white), and maternal age (+/−2 years). However, information on the county of residence is unavailable in the data, which we downloaded from the CEDR database. Therefore, we generated a new variable on DOE sites as surrogate variable based on birth places of the study subject. In the CEDR database, the birth places were divided into the following eight categories: Hanford hospitals, Idaho hospitals, Tennessee hospitals, home, birth center, maternity hospitals and unknown. Those who were born at home, or in birth centers, maternity hospitals and unknown were coded as a missing value in the variable on DOE sites (23 and 8 subjects in the original study and present study, respectively).

In the available data set, the ID number to identify the matched control(s) for each case was unavailable; therefore, we could not conduct conditional logistic models. Therefore, we conducted conventional logistic analysis. When the analysis of matched case-control data ignores case-control matching, all the matched factors should be treated as potential confounders in statistical analysis [21]. Therefore, we adjusted for the matching variables (birth year, county of residence, sex, ethnicity and maternal age). In addition, we also included GA, DOE sites and age at diagnosis as independent variables in the logistic model as well. Age at diagnosis for controls was calculated, using the year of diagnosis, which was assigned to the controls by the original study (the year of diagnosis of each case was assigned to the corresponding controls by the original study). The DOE sites were used as a surrogate variable for the county of residence.

Low BW is defined, by the World Health Organization, as a BW smaller than 2500 g. High BW is defined by Centers for Disease Control and Prevention as a BW larger than 4000 g [22]. Furthermore, we used BW corrected for GA to categorize the subjects as being LGA, AGA and SGA. In the present study, LGA children were those with BWs greater than the 90th percentile for their GAs. Children whose BW was below the 10th percentile for their GAs were classified as SGA. AGA children were those whose BWs were in the 10–90 percentile for their GAs. Those categories were constructed, using the US national reference for fetal growth [23].

## Results

The characteristics of the CNS tumor and leukemia cases and the controls, according to the factors matched (or surrogate factors) in the original study, are presented in Table 1. Cases and controls showed similar distributions regarding those factors. One exception was the year of birth. CNS tumor cases did not have those born before 1952. The proportion of children with CNS tumors born in later years, especially after 1970, was higher compared to that of children with leukemia. In this table, DOE sites are a surrogate factor for the county of residence, which was matched in the original study, but was unavailable in the database. Regarding the DOE sites’ distribution, the control group had more subjects in Hanford and less in Oak Ridge. In order to control those potential confounders, we included those variables in the conventional logistic models in the risk analysis.

**Table 1:** Characteristics of cases and controls by factors matched (or surrogate factors) in the original study

Variables	Controls	Cases	
		CNS tumor	Leukemia
	(*N* = 822)	(*N* = 72)	(*N* = 124)
Year of birth	1946–1989	1952–1989	1949–1989
1946–1959	120 (14.6%)	11 (15.3%)	22 (17.7%)
1960–1969	240 (29.2%)	16 (22.2%)	44 (35.5%)
1970–1979	294 (35.8%)	28 (38.9%)	35 (28.2%)
1980–1989	168 (20.4%)	17 (23.6%)	23 (18.6%)
Age at diagnosis (years)^a^			
Mean (SD)	6.1 (4.4)	5.6 (4.3)	5.3 (4.1)
Min-Max	0–15	0–14	0–14
Sex			
Male	493 (60.0%)	45 (62.5%)	71 (57.3%)
Female	329 (40.0%)	27 (37.5%)	53 (42.7%)
Ethnicity			
Black	18 (2.2%)	3 (4.2%)	2 (1.6%)
White	804 (97.8%)	69 (95.8%)	122 (98.4%)
Maternal age (years)			
Mean (SD)	25.5 (5.4)	25.2 (5.2)	25.5 (5.8)
Min-Max	14–44	15–37	16–42
DOE sites^b^			
Hanford	271 (32.9%)	19 (26.4%)	28 (22.6%)
INEL	183 (22.3%)	15 (20.8%)	33 (26.6%)
Oak Ridge	363 (44.2%)	37 (51.4%)	61 (49.2%)
Unknown	5 (0.6%)	1 (1.4%)	2 (1.6%)
Gestational age (weeks)^c^			
Mean (SD)	39.3 (1.9)	38.5 (2.4)	39.4 (1.8)
Min-Max	28–45	27–43	34–44

*SD* standard deviation, *DOE* Department of Energy

^a^ Age at diagnosis for controls was calculated, using the year of diagnosis assigned by the original study, which was matched case-control study

^b^ DOE sites: a surrogate variable for county of residence

^c^ Not matched in the original study

In the following tables, the results of the logistic analysis are summarized. The analysis for leukemia risk was also conducted and their results are included in those tables for comparison. As shown in Table 2, CNS tumor risk increased with BW (*p* value for trend =0.010). When those with BW less than 2500 g were excluded, the association became stronger (*p* for trend <0.001). Even among those in the normal-BW range (2500–4000 g), the p for trend was significant (*p* = 0.012). The increasing trend was mainly from those larger than 4000 g. The OR for this high BW adjusted for GA was 2.5 (95%CI = 1.2, 5.2) when compared to normal BW (2500–4000 g). The GA-unadjusted OR was 2.0 (95%CI = 1.0, 4.1) (Additional file 1: Table S1). In this table, we also made a comparison between low-BW and normal-BW children. The CNS tumor risk was also increased among low-BW children, and the OR was 2.0 (95%CI = 0.7–5.9); however, the increase was not statistically significant (*p* = 0.241).

**Table 2:** The association between birth weight and the risk of CNS tumors

Birth weight	Controls	CNS tumors	OR	95%CI		*P* value
				Lower	Upper	
Total subjects						
< 2500 g	24	7	1.8	0.5	5.8	0.363
2500- < 3000 g	137	7	0.6	0.2	1.4	0.214
3000- < 3500 g	305	21	1	Reference		
3500–4000 g	276	25	1.5	0.8	2.8	0.205
> 4000 g	80	12	2.9	1.3	6.6	0.012
			*P for homogeneity = 0.017* *For all: P for trend = 0.010 (beta = 0.0007)* *For birth weight ≥ 2500 g:P for trend < 0.001 (beta = 0.0011)* *For birth weight 2500–4000 g: P for trend = 0.012 (beta = 0.0011)*			
< 2500 g	24	7	2.0	0.7	5.9	0.241
2500–4000 g	718	53	1	Reference		
> 4000 g	80	12	2.5	1.2	5.2	0.018
			*P for homogeneity = 0.028*			
The risk of high-birth-weight and LGA children compared to normal-birth-weight children^a^						
2500–4000 g	718	53	1	Reference		
> 4000 g and LGA	48	8	2.7	1.1	6.2	0.035
The risk of high-birth-weight and SGA/AGA children compared to normal-birth-weight children^a^						
2500–4000 g	718	53	1	Reference		
> 4000 g and SGA/AGA	32	4	2.2	0.7	6.7	0.209

*LGA* large for gestational age, *SGA* small for gestational age, *AGA* appropriate for gestational age

ORs and corresponding 95%CIs and p values were adjusted for sex, ethnicity, year of birth, age at diagnosis, gestational age (continuous variable), maternal age and DOE sites

^a^ Children with low-birth weight were not included in the analyses

Among the high-BW children, SGA, AGA and LGA accounted for 2, 33 and 51 children, respectively. When high-BW children were restricted to LGA, the OR for CNS tumors was 2.7 (95%CI = 1.1, 6.2; *p* = 0.035) as shown in the middle panel of Table 2. When high-BW children were restricted to SGA/AGA (the lower panel of Table 2), the OR for CNS tumors became smaller (OR = 2.2; 95%CI = 0.7, 6.7; *p* = 0.209).

Leukemia risk was not associated with BW (Table 3). In the lower panel of Table 3, among high-BW children, the risk was increased by 40%, but the increase was not statistically significant.

**Table 3:** The association between birth weight and the risk of leukemia

Birth weight	Controls	Leukemia cases	OR	95%CI	*P* value	
				Lower	Upper	
Total subjects						
< 2500 g	24	3	0.7	0.2	2.6	0.564
2500- < 3000 g	137	20	0.7	0.4	1.3	0.300
3000- < 3500 g	305	54	1	Reference		
3500–4000 g	276	33	0.7	0.4	1.1	0.092
> 4000 g	80	14	1.1	0.6	2.2	0.752
			*P for homogeneity = 0.396* *For all: P for trend = 0.778 (beta = 0.00006)* *For birth weight ≥ 2500 g: P for trend = 0833 (beta = 0.00005)* *For birth weight 2500–4000 g: P for trend = 0.475 (beta = −0.00022)*			
< 2500 g	24	3	0.8	0.2	3.0	0.765
2500–4000 g	718	107	1	Reference		
> 4000 g	80	14	1.4	0.7	2.6	0.343
			*P for homogeneity = 0.611*			
The risk of high-birth-weight and LGA children compared to normal-birth-weight children^a^						
2500–4000 g	718	107	1	Reference		
> 4000 g and LGA	48	10	1.7	0.8	3.7	0.166
The risk of high-birth-weight and SGA/AGA children compared to normal-birth-weight children^a^						
2500–4000 g	718	107	*1*	*Reference*		
> 4000 g and SGA/AGA	32	4	*0.9*	*0.3*	*2.7*	*0.865*

*LGA* large for gestational age, *SGA* small for gestational age, *AGA* appropriate for gestational age

ORs and corresponding 95%CIs and p values were adjusted for sex, ethnicity, year of birth, age at diagnosis, gestational age (continuous variable), maternal age and DOE sites

^a^ Children with low-birth weight were not included in the analyses

We examined the association of GA with the risks of CNS tumor and leukemia (Table 4). The CNS tumor risk was inversely associated with longer GA (42 weeks or longer) after adjustment for BW (p for trend = 0.001). However, the leukemia risk was elevated among children with longer GA.

**Table 4:** The association between gestational age and the risks of CNS tumors and leukemia

Gestational age	Controls	Cases	OR	95%CI		*P* value
				Lower	Upper	
For the analysis of CNS tumor risk						
< 37 weeks	54	9	1.5	0.6	3.9	0.405
37–39 weeks	331	38	1	Reference		
40–41 weeks	366	20	0.3	0.2	0.6	<0.001
> 41 weeks	71	5	0.4	0.1	1.0	0.048
						*P for trend = 0.001*
For the analysis of Leukemia risk						
< 37 weeks	54	6	0.9	0.3	2.4	0.842
37–39 weeks	331	51	1	Reference		
40–41 weeks	366	52	0.7	0.5	1.2	0.175
> 41 weeks	71	15	1.2	0.6	2.3	0.659
						*P for trend = 0.930*

ORs and corresponding 95%CIs and p values were adjusted for sex, ethnicity, year of birth, age at diagnosis, maternal age, birth weight (5-category variable) and DOE sites

We examined the association of LGA and SGA with the risks of CNS tumors and leukemia (Tables 5 and 6). LGA children were at higher risks of CNS tumors and leukemia, but neither increase was statistically significant. Even when the subjects were limited to those with BWs 2500 g or larger, or those with BWs 3000 g or larger, the results did not change sizably. The risk of CNS tumors or leukemia was not statistically significantly associated with SGA.

**Table 5:** CNS tumor risk among small-for-gestational-age and large-for-gestational-age children

Birth weight	Controls	CNS tumors	OR	95%CI		*P* value
				Lower	Upper	
Total subjects						
SGA	189	15	0.9	0.4	1.7	0.643
AGA	566	48	1	Reference		
LGA	63	9	1.8	0.8	3.9	0.163
						*P for homogeneity = 0.307*
Birth weight 2500 g or larger						
SGA	177	12	0.8	0.4	1.6	*0.494*
AGA	544	44	1	Reference		
LGA	63	9	2.0	0.9	4.5	*0.101*
						*P for homogeneity = 0.173*
Birth weight 3000 g or larger						
SGA	97	9	1.2	0.5	3.1	*0.672*
AGA	497	40	1	Reference		
LGA	63	9	2.0	0.9	4.4	*0.113*
						*P for homogeneity = 0.279*

*SGA* small for gestational age, *AGA* appropriate for gestational age, *LGA* large for gestational age

ORs and corresponding 95%CIs and p values were adjusted for sex, ethnicity, year of birth, age at diagnosis, maternal age and DOE sites

**Table 6:** Leukemia risk among small-for-gestational-age and large-for-gestational-age children

Birth weight	Controls	Leukemia cases	OR	95%CI		*P* value
				Lower	Upper	
Total subjects						
SGA	189	30	0.9	0.6	1.5	0.696
AGA	566	83	1	Reference		
LGA	63	11	1.4	0.7	2.9	0.342
						*P for homogeneity = 0.555*
Birth weight 2500 g or larger						
SGA	177	29	0.9	0.5	1.5	0.714
AGA	554	81	1	Reference		
LGA	63	11	1.4	0.7	2.9	0.340
						*P for homogeneity = 0.561*
Birth weight 3000 g or larger						
SGA	97	18	0.9	0.5	1.8	0.841
AGA	497	72	1	Reference		
LGA	63	11	1.5	0.7	3.1	0.298
						*P for homogeneity = 0.547*

*SGA* small for gestational age, *AGA* appropriate for gestational age, *LGA* large for gestational age

ORs and corresponding 95%CIs and p values were adjusted for sex, ethnicity, year of birth, age at diagnosis, maternal age and DOE sites

The American Congress of Obstetricians and Gynecologists has redefined “term pregnancy” and replaced it with four new definitions of “term” deliveries: early term (37 weeks 0 day - 38 weeks 6 days), full term (39 weeks 0 day - 40 weeks 6 days), late term (41 weeks 0 day - 41 weeks 6 days) and post term (42 weeks 0 day and beyond). We relaxed the definition for normal GA to avoid losing the number of cases, and used children with GA of 37–42 weeks. This decision increased the number of CNS tumor and leukemia cases, and the controls by 5, 11 and 51, respectively. However, the associations of BW or LGA/SGA with the risk of CNS tumors or leukemia did not change appreciably (Additional file 2: Table S2, Additional file 3: Table S3 and Additional file 4: Table S4).

## Discussion

The present study showed that higher BW was positively associated with childhood CNS tumor risk with or without adjustment for GA. This observed association was mainly from those larger than 4000 g. The OR among the high-BW children was 2.5 (95%CI = 1.2, 5.2) with adjustment for GA, and 2.0 (95%CI = 1.0, 4.1) without adjustment. Those values are higher than those reported by the previously conducted studies [6, 9, 11, 12].

Leukemia risk was increased (OR = 1.4; 95%CI = 0.7, 2.6; *p* = 0.343) among the high-BW children. A meta-analysis reported a similar OR (OR = 1.35; 95%CI = 1.24, 1.48) on the basis of 32 studies [7]. The fact that this study was unable to establish a significant association between high BW and leukemia risk could be attributed to the fact that the effect estimate of high BW might be too small, relative to the sample size.

Even in the normal-BW range (2500–4000 g), higher BW was still positively associated with childhood CNS tumor risk (p for trend = 0.012), but not with leukemia risk (p for trend = 0.475). To date, no study has found that BW is related to CNS tumor risk in the normal-BW range. However, several studies examined the association of BW itself with CNS tumor risk. The magnitude of the OR change per 1000-g BW obtained from the present study was similar to those reported by other studies [5, 6, 9, 13].

In the present study, GA was inversely associated with CNS tumor risk (p for trend = 0.001). This finding is at variance with those obtained from the other studies, which reported a weak positive association between BW and CNS tumor risk [6, 14, 15]. The association between leukemia risk and GA was not found in our study (p for trend = 0.930) as was the case with the other studies [6, 16].

BW and GA are known to be closely related to each other [17]. When the high-BW children were restricted to those who were LGA, the OR was 2.7 (95%CI = 1.1, 6.2). When high-BW children were restricted to those without LGA, the OR was 2.2 (95%CI = 0.7, 6.7), which is smaller than the OR for high-BW and LGA children. In the present study, SGA was not statistically related to the risk of CNS tumors or leukemia.

Our study found an increased risk of CNS tumors among LGA children, but the increase was not statistically significant. The OR obtained in our study (OR = 1.8; 95%CI = 0.8, 3.9), which was larger than those reported by the other studies (in which the ORs were in the range of 1.09–1.18) [6, 9, 11, 12]. In the case of leukemia, our study obtained an OR of 1.4 (95%CI = 0.7, 2.9), which is similar to those reported by other studies (in which the ORs were in the range of 1.45–1.66) [6, 11].

In the present study, CNS tumor risk was not associated with SGA (OR = 0.9; 95%CI = 0.4, 1.7) as was the case with the other studies [6, 9, 11, 12]. The OR for leukemia was 0.9 (95%CI = 0.6, 1.5). The association between leukemia risk and SGA on the literature is inconsistent. The ORs obtained from the US and German studies were in the range of 0.78 to 1.00 [6, 11], and were 1.2 to 1.8 in Nordic study [12]. Our result is similar to the values reported by the Texan and German studies [6, 11].

CNS tumors have various histological types which may have different etiological backgrounds. The three most common types of childhood CNS tumors include medulloblastomas, astrocytomas and malignant gliomas, which accounted for 50% of those tumors in a US study [24]. A meta-analysis of eight studies reported in 2008 showed that high-BW children had slightly elevated risks of astrocytoma (OR = 1.38, 95%CI = 1.07, 1.79) and medulloblastoma (OR = 1.27, 95%CI = 1.02, 1.60) [10]. Among the eight studies, only California study considered the GA as a potential confounder [15, 25–31]. In the present study, we did not have information on the pathological types of the tumors.

Several mechanisms which stimulate prenatal weight gain and act simultaneously as long-term carcinogens might explain the association between high BW and the increased risk of CNS tumors. First, high BW could be an indicator of a greater number of cells, leading to more cell divisions. It is strongly suspected that such a condition could make them more vulnerable to carcinogenic agents and therefore, the cancer risk increases after birth [32]. BW is known to be positively correlated with insulin-like growth factor-1, which is strongly suggested to be involved in brain ontogenesis and carcinogenesis [33, 34]. Second, Heuch et al. [27] proposed the involvement of excess prenatal nutrition in medulloblastoma development, and suspected that high BW is an important indicator of excess nutrition in the last gestational trimester. They suspected that ample nutrition may interfere with the migration of granular neuronal cells, which starts at approximately 30 gestational weeks. If the cells migrate incompletely, they may remain immature. As a result, neoplastic potential of the cell may increase.

In the present study, childhood cancer patients were diagnosed from 1957 to 1991. As shown in Table 1, the proportion of CNS tumor patients seems to have increased with calendar year, though this upward trend was not observed in the case of childhood leukemia. The improvement in diagnostic technologies could have led to artifactual increases in the rate CNS tumor occurrence [35]. It is to be noted that computed tomography and magnetic resonance imaging scans were widely used in the 1970s and 1980s, respectively.

Our study has several limitations. First, the results should be treated with considerable caution because of the limited number of cases. Regarding the leukemia risk, we failed to find a significant association. The effect estimate of high BW might be too small compared to the sample size. Second, cases were ascertained mainly from hospitals. Although the original study described “cancer registry” as a source of case ascertainment, we assumed that this might have been a hospital-based registry, as population-based cancer registries were unavailable in the 1957–1991 period. Thus, we could deny the possibility that cases without consultation at the hospitals or diagnosed outside of the study areas could be missed. Third, we lacked information on the subtypes of CNS tumors and leukemia. Typically, tumor registries did not cover those years. Death certificates did not provide identification of a hospital where diagnostic information might be located. The data in hospital records were insufficient for those years. Fourth, the study encountered problems in obtaining the birth records of the cases and controls. While Sever et al. received high level of cooperation from many hospitals that provided them with access to records, the medical records themselves were often missing and the data were incomplete [18]. Since these problems were mainly with newborn records, that they did not affect the cases and controls differently. Fifth, the study did not collect sufficient information on the socio-economic status (SES) of the subjects. Unlike in the case of the relationship between SES and low BW, the association between SES and high BW risk is not consistent [36]. Many studies have been conducted to examine the association between SES and leukemia risk. On reviewing studies published until 1982, higher SES was suspected to be related to childhood leukemia risk [37]. A review by Poole et al. [38], however, noted that most later studies consistently reported inverse associations of childhood leukemia with SES; it was concluded, therefore, that associations between SES measures and childhood leukemia likely vary with the time and place. A study based on 5240 leukemia cases from the Canadian cancer registries, that covered at least 95% of all the cases, reported a slightly lower relative risk of leukemia in the poorest group (RR = 0.87; 95%CI = 0.80, 0.95) [39]. A similar finding was also reported in a large case-control study from the UK (OR = 0.99, 95%CI = 0.96, 1.01) [40]. Thus, the effect of SES on the association between BW and leukemia risk may be considerably small even if SES is a potential confounding factor. The association between SES and CNS tumor risk was still inclusive [41–44]. Sixth, information on maternal comorbidities was not available in this data set. Although gestational diabetes mellitus is the most important risk factor for high BW and LGA, we could not examine the effect of gestational diabetes mellitus on childhood cancer risk. Finally, SGA was not a risk factor for childhood cancers in our study. The Barker hypothesis shows that low BW is associated to the risk of developing chronic diseases in later life [45–47]. However, the association of low BW and childhood cancer risk has not been clarified.

## Conclusion

High-BW and LGA children had an elevated childhood CNS tumor risk. In the normal BW range, BW itself was positively related to CNS tumor risk. Low BW was not associated with an increased CNS tumor risk. No significant association between BW and childhood leukemia risk was observed in this study.

## Additional files

Additional file 1: Table S1. The association between birth weight and CNS tumor risk without adjustment for gestational age. The GA-unadjusted OR for high BW was 2.5 (95%CI = 1.2, 5.2) when compared to normal BW (2500–4000 g). (DOCX 21 kb)

Additional file 2: Table S2. The association between birth weight and the CNS tumor risk among children with gestational age of 37–42 weeks**.** When compared to the results in Table 2, the ORs and 95%CIs for high or low BW did not change appreciably. (DOCX 24 kb)

Additional file 3: Table S3. The association between birth weight and leukemia risk among children with gestational age of 37–42 weeks. When compared to the results in Table 3, the ORs and 95%CIs for high or low BW did not change appreciably. (DOCX 23 kb)

Additional file 4: Table S4. The risk of CNS tumors or leukemia among small-for-gestational-age and large-for-gestational-age children with gestational age of 37–42 weeks. When compared to the results in Tables 5 and 6, the ORs for LGA/SGA did not change appreciably. (DOCX 27 kb)

## Abbreviations

AGA: Appropriate for gestational age
ALL: Acute lymphoblastic1 leukemia
AML: Acute myeloid leukemia
BW: Birth weight
CEDR: Comprehensive epidemiologic data resource
CI: Confidence interval
CNS: Central nervous system
DOE: Department of energy
GA: Gestational age
LGA: Large for gestational age
OR: Odds ratio
SES: Socio-economic status
SGA: Small for gestational age
