# Supplementary material for: The association between high birth weight and the risks of childhood CNS tumors and leukemia: an analysis of a US case-control study in an epidemiological database
Source: BMC Cancer. 2017 Oct 16;17:687. doi: 10.1186/s12885-017-3681-y (PMC5644053; doi:10.1186/s12885-017-3681-y)
Supplement: Supplementary file 1 — The association between birth weight and CNS tumor risk without adjustment for gestational age. The GA-unadjusted OR for high BW was 2.5 (95%CI = 1.2, 5.2) when compared to normal BW (2500–4000 g). (DOCX 21 kb) [file 12885_2017_3681_MOESM1_ESM.docx]

**Additional table 1. The association between birth weight and CNS tumor risk without adjustment for gestational age**

| **Birthweight** | **Controls** | **CNS tumors** | **OR** | **95%CI** | | **P value** |
| --- | --- | --- | --- | --- | --- | --- |
|  |  |  |  | **Lower** | **Upper** |  |
| <2,500 g | 24 | 7 | 4.1 | 1.6 | 10.1 | 0.007 |
| 2,500-4,000 g | 718 | 53 | 1 | Reference | |  |
| >4,000 g | 80 | 12 | 2.0 | 1.0 | 4.1 | 0.058 |
|  |  |  |  |  |  | *P for homogeneity=0.006* |
| ORs and corresponding 95%CIs and p values were adjusted for sex, ethnicity, year of birth, age at diagnosis, maternal age and DOE sites. | | | | | | |
