# Supplementary material for: The association between high birth weight and the risks of childhood CNS tumors and leukemia: an analysis of a US case-control study in an epidemiological database
Source: BMC Cancer. 2017 Oct 16;17:687. doi: 10.1186/s12885-017-3681-y (PMC5644053; doi:10.1186/s12885-017-3681-y)
Supplement: Supplementary file 2 — The association between birth weight and the CNS tumor risk among children with gestational age of 37–42 weeks. When compared to the results in Table 2, the ORs and 95%CIs for high or low BW did not change appreciably. (DOCX 24 kb) [file 12885_2017_3681_MOESM2_ESM.docx]

**Additional table 2. The association between birth weight and the CNS tumor risk among children with gestational age of 37-42 weeks**

| **Birthweight** | **Controls** | **CNS tumors** | **OR** | **95%CI** | | **P value** |
| --- | --- | --- | --- | --- | --- | --- |
|  |  |  |  | **Lower** | **Upper** |  |
| **Total subjects** |  |  |  |  |  |  |
| <2,500 g | 11 | 3 | 3.8 | 0.9 | 15.7 | 0.091 |
| 2,500-<3,000 g | 112 | 5 | 0.6 | 0.2 | 1.6 | 0.251 |
| 3,000-<3,500 g | 288 | 19 | 1 | Reference | |  |
| 3,500-4,000 g | 262 | 24 | 1.7 | 0.9 | 3.3 | 0.099 |
| >4,000 g | 75 | 11 | 3.6 | 1.5 | 8.7 | 0.005 |
|  |  |  | *P for homogeneity=0.007*  *P for trend=0.012*  *P for trend=0.001 (birth weight ≥2,500 g)*  *P for trend=0.019 (birth weight 2,500-4,000 g)* | | | |
| <2,500 g | 11 | 3 | 3.4 | 0.9 | 13.2 | 0.111 |
| 2,500-4,000 g | 662 | 48 | 1 | Reference | |  |
| >4,000 g | 75 | 11 | 2.8 | 1.3 | 5.9 | 0.014 |
|  |  |  | *P for homogeneity=0.016* | | | |
| **The risk of high-birth-weight and LGA children compared to normal-birth-weight children*** | | | | | | |
| 2,500-4,000 g | 662 | 48 | 1 | Reference |  |  |
| >4,000 g and LGA | 44 | 7 | 2.7 | 1.1 | 6.7 | 0.048 |
|  |  |  |  |  |  |  |
| **The risk of high-birth-weight and SGA/AGA children compared to normal-birth-weight children*** | | | | | | |
| 2,500-4,000 g | 662 | 48 | 1 | Reference |  |  |
| >4,000 g and GA/AGA | 31 | 4 | 2.9 | 0.9 | 9.6 | 0.098 |
| LGA: large for gestational age, SGA: small for gestational age, AGA: appropriate for gestational age | | | | | | |
| ORs and corresponding 95%CIs and p values were adjusted for sex, ethnicity, year of birth, age at diagnosis, gestational age (continuous variable), maternal age and DOE sites.  * Children with low-birth weight were not included in the analyses | | | | | | |
