# Supplementary material for: The association between high birth weight and the risks of childhood CNS tumors and leukemia: an analysis of a US case-control study in an epidemiological database
Source: BMC Cancer. 2017 Oct 16;17:687. doi: 10.1186/s12885-017-3681-y (PMC5644053; doi:10.1186/s12885-017-3681-y)
Supplement: Supplementary file 3 — The association between birth weight and leukemia risk among children with gestational age of 37–42 weeks. When compared to the results in Table 3, the ORs and 95%CIs for high or low BW did not change appreciably. (DOCX 23 kb) [file 12885_2017_3681_MOESM3_ESM.docx]

**Additional table 3. The association between birth weight and leukemia risk among children with gestational age of 37-42 weeks**

| **Birthweight** | **Controls** | **Leukemia cases** | **OR** | **95%CI** | | **P value** |
| --- | --- | --- | --- | --- | --- | --- |
|  |  |  |  | **Lower** | **Upper** |  |
| <2,500 g | 11 | 1 | 0.5 | 0.1 | 3.7 | 0.413 |
| 2,500-<3,000 g | 112 | 18 | 0.8 | 0.4 | 1.4 | 0.425 |
| 3,000-<3,500 g | 288 | 51 | 1 | Reference | |  |
| 3,500-4,000 g | 262 | 30 | 0.7 | 0.4 | 1.1 | 0.106 |
| >4,000 g | 75 | 14 | 1.3 | 0.7 | 2.7 | 0.429 |
|  |  |  | *P for homogeneity=0.275*  *P for trend=0.715*  *P for trend =0.314 (birth weight 2,500-4,000 g)* | | | |
| <2,500 g | 11 | 1 | 0.5 | 0.1 | 4.4 | 0.529 |
| 2,500-4,000 g | 662 | 99 | 1 | Reference | |  |
| >4,000 g | 75 | 14 | 1.6 | 0.8 | 3.1 | 0.158 |
|  |  |  | *P for homogeneity=0.296* | | | |
| LGA: large for gestational age, SGA: small for gestational age, AGA: appropriate for gestational age | | | | | | |
| ORs and corresponding 95%CIs and p values were adjusted for sex, ethnicity, year of birth, age at diagnosis, gestational age (continuous variable), maternal age and DOE sites. | | | | | | |
