# Supplementary material for: The association between high birth weight and the risks of childhood CNS tumors and leukemia: an analysis of a US case-control study in an epidemiological database
Source: BMC Cancer. 2017 Oct 16;17:687. doi: 10.1186/s12885-017-3681-y (PMC5644053; doi:10.1186/s12885-017-3681-y)
Supplement: Supplementary file 4 — The risk of CNS tumors or leukemia among small-for-gestational-age and large-for-gestational-age children with gestational age of 37–42 weeks. When compared to the results in Tables 5 and 6, the ORs for LGA/SGA did not change appreciably. (DOCX 27 kb) [file 12885_2017_3681_MOESM4_ESM.docx]

**Additional table 4. The risk of CNS tumors or leukemia among small-for-gestational-age and large-for-gestational-age children with gestational age of 37-42 weeks**

| **Birth weight** | **Controls** | **Cases** | **OR** | **95%CI** | | **P value** |
| --- | --- | --- | --- | --- | --- | --- |
|  |  |  |  | **Lower** | **Upper** |  |
| **CNS tumors** |  |  |  |  |  |  |
| **Total subjects** |  |  |  |  |  |  |
| SGA | 186 | 15 | 0.9 | 0.5 | 2.0 | 0.963 |
| AGA | 507 | 39 | 1 | Reference | |  |
| LGA | 55 | 8 | 2.0 | 0.9 | 4.7 | 0.119 |
|  |  |  |  |  |  | *P for homogeneity=0.286* |
| **Birth weight 2,500 g or larger** | | |  |  |  |  |
| SGA | 175 | 12 | 0.8 | 0.4 | 1.7 | *0.563* |
| AGA | 507 | 39 | 1 | Reference | |  |
| LGA | 55 | 8 | 2.1 | 0.9 | 4.8 | *0.113* |
|  |  |  |  |  |  | *P for homogeneity=0.207* |
| **Birth weight 3,000 g or larger** | | |  |  |  |  |
| SGA | 97 | 9 | 1.1 | 0.4 | 2.9 | *0.797* |
| AGA | 473 | 37 | 1 | Reference | |  |
| LGA | 55 | 8 | 2.0 | 0.9 | 4.7 | *0.131* |
|  |  |  |  |  |  | *P for homogeneity=0.319* |
| **LEUKEMIA** |  |  |  |  |  |  |
| **Total subjects** |  |  |  |  |  |  |
| SGA | 186 | 30 | 0.9 | 0.6 | 1.5 | 0.752 |
| AGA | 507 | 73 | 1 | Reference | |  |
| LGA | 55 | 11 | 1.8 | 0.8 | 3.7 | 0.148 |
|  |  |  |  |  |  | *P for homogeneity=0.306* |
| **Birth weight 2,500 g or larger** | | |  |  |  |  |
| SGA | 175 | 29 | 0.9 | 0.6 | 1.6 | 0.812 |
| AGA | 507 | 73 | 1 | Reference | |  |
| LGA | 55 | 11 | 1.7 | 0.8 | 3.6 | 0.157 |
|  |  |  |  |  |  | *P for homogeneity=0.334* |
| **Birth weight 3,000 g or larger** | | |  |  |  |  |
| SGA | 97 | 18 | 0.9 | 0.5 | 1.9 | 0.918 |
| AGA | 473 | 66 | 1 | Reference | |  |
| LGA | 55 | 11 | 1.9 | 0.9 | 4.0 | 0.112 |
|  |  |  |  |  |  | *P for homogeneity=0.267* |
| SGA: Small for gestational age, AGA: Appropriate for gestational age, LGA: Large for gestational age | | | | | | |
| ORs and corresponding 95%CIs and p values were adjusted for sex, ethnicity, year of birth, age at diagnosis, maternal age and DOE sites. | | | | | | |
